# Correction: Synthesis, characterization and biological studies of pyrazole-linked Schiff bases and their copper(ii) complexes as potential therapeutics

**DOI:** 10.1039/d5ra90134k

**Published:** 2025-11-18

**Authors:** Pratima Kumari, Aman Kumar, Ramesh Kataria, Naveen Kumar Kaushik, Mukhtar Ahmed, Azaj Ansari, Ekta, Mettle Brahma, Mulaka Maruthi, Yangala Sudheer Babu, Bijender Singh, Vinod Kumar

**Affiliations:** a Department of Chemistry, School of Basic Sciences, Central University of Haryana Mahendergarh-123031 Haryana India vinodkumar@cuh.ac.in; b Department of Chemistry, Panjab University Chandigarh-160014 India rkataria@pu.ac.in; c Amity Institute of Virology and Immunology, Amity University Noida-201313 Uttar Pradesh India; d Department of Biochemistry, School of Interdisciplinary and Applied Sciences, Central University of Haryana Mahendergarh-123031 Haryana India; e Department of Biotechnology, School of Interdisciplinary and Applied Sciences, Central University of Haryana Mahendergarh-123031 Haryana India; f Department of Industrial Biotechnology, College of Biotechnology, Chaudhary Charan Singh Agriculture University Hisar 125004 Haryana India

## Abstract

Correction for “Synthesis, characterization and biological studies of pyrazole-linked Schiff bases and their copper(ii) complexes as potential therapeutics” by Pratima Kumari *et al.*, *RSC Adv.*, 2025, **15**, 42299–42314, https://doi.org/10.1039/D5RA06008G.

The authors regret that in the original article the name and affiliation details for author Naveen Kumar Kaushik were incorrect. The correct name and affiliation information can be found herein.

Additionally, the authors note that an incorrect column header was used to label data in Table 1 of the original article. Column header ‘% Parasitemia’ should be ‘% Growth (parasitaemia)’ as seen in the revised Table 1 here.

**Table 1:** *In vitro* antimalarial activity of the compounds against *P. falciparum* (Pf3D7) after 72 h

Compounds	% Growth (parasitaemia)		% Suppression	
	12.5 µg mL^−1^	100 µg mL^−1^	12.5 µg mL^−1^	100 µg mL^−1^
**3a**	93.33	**2.95**	6.67	**97.05**
**3b**	68.38	62.57	31.62	37.43
**3c**	52.90	5.81	47.10	**94.19**
**3d**	42.19	**2.47**	57.81	**97.53**
**3e**	73.81	37.62	26.19	62.38
**4a**	**2.0**	**0.0**	**98.0**	**100.0**
**4b**	21.71	**1.90**	78.29	**98.10**
**4c**	23.62	**1.90**	78.29	**98.10**
**4d**	**0.0**	**0.0**	**100.0**	**100.0**
**4e**	**0.0**	**0.0**	**100.0**	**100.0**
CQ	**0.95**	**0.95**	**99.05**	**99.05**

The Royal Society of Chemistry apologises for these errors and any consequent inconvenience to authors and readers.

